# Simulated deep CT characterization of liver metastases with high-resolution filtered back projection reconstruction

**DOI:** 10.1186/s42492-024-00161-y

**Published:** 2024-06-11

**Authors:** Christopher Wiedeman, Peter Lorraine, Ge Wang, Richard Do, Amber Simpson, Jacob Peoples, Bruno De Man

**Affiliations:** 1 Department of Electrical and Computer Engineering, Rensselaer Polytechnic Institute, Troy, NY 12180 USA; 2 GE Research - Healthcare, Niskayuna, NY 12309 USA; 3 Department of Biomedical Engineering, Rensselaer Polytechnic Institute, Troy, NY 12180 USA; 4 Department of Radiology, Memorial Sloan Kettering Cancer Center, New York, NY 10065 USA; 5 Biomedical Computing and Informatics, Queen’s University, Kingston, ON K7L 3N6 Canada

**Keywords:** Radiomics, Deep learning, Computed tomography, Colorectal liver metastases, Virtual clinical trials, Image reconstruction

## Abstract

Early diagnosis and accurate prognosis of colorectal cancer is critical for determining optimal treatment plans and maximizing patient outcomes, especially as the disease progresses into liver metastases. Computed tomography (CT) is a frontline tool for this task; however, the preservation of predictive radiomic features is highly dependent on the scanning protocol and reconstruction algorithm. We hypothesized that image reconstruction with a high-frequency kernel could result in a better characterization of liver metastases features via deep neural networks. This kernel produces images that appear noisier but preserve more sinogram information. A simulation pipeline was developed to study the effects of imaging parameters on the ability to characterize the features of liver metastases. This pipeline utilizes a fractal approach to generate a diverse population of shapes representing virtual metastases, and then it superimposes them on a realistic CT liver region to perform a virtual CT scan using CatSim. Datasets of 10,000 liver metastases were generated, scanned, and reconstructed using either standard or high-frequency kernels. These data were used to train and validate deep neural networks to recover crafted metastases characteristics, such as internal heterogeneity, edge sharpness, and edge fractal dimension. In the absence of noise, models scored, on average, 12.2% ($\alpha =0.012$) and 7.5% ($\alpha =0.049)$ lower squared error for characterizing edge sharpness and fractal dimension, respectively, when using high-frequency reconstructions compared to standard. However, the differences in performance were statistically insignificant when a typical level of CT noise was simulated in the clinical scan. Our results suggest that high-frequency reconstruction kernels can better preserve information for downstream artificial intelligence-based radiomic characterization, provided that noise is limited. Future work should investigate the information-preserving kernels in datasets with clinical labels.

## Introduction

Over the past decade, colorectal cancer has become an increasingly prominent medical challenge. Contributing annually to approximately 50,000 deaths in the United States and 900,000 worldwide, it is now the third most commonly diagnosed cancer in the United States and is considered the fourth deadliest cancer. Diseased patients often die from colorectal liver metastases (CRLM) rather than from the primary cancer. Although a multitude of treatment options exist, including resection, chemotherapy, and ablation, monitoring patient responses and choosing the most effective therapies is a complex problem requiring further research [1–3].

Accurate and continuous monitoring of disease progression and CRLM is critical for optimizing patient outcomes. To this end, X-ray computed tomography (CT) is one of the best and most widely available imaging modalities for observing CRLM progression. Imaging features such as the largest metastasis (met) diameter and number of hepatic mets have been investigated as prognostic markers [4]. In addition, CT image texture analysis of hepatic mets and the entire liver has been studied to predict treatment response [5–8]. Broadly speaking, the fractal dimension of CT tumor images has been investigated as a prognostic feature in a variety of oncological contexts, including response prediction to chemoradiation therapies in patients with locally advanced rectal cancer and survival prediction in sunitinib treated patients with hepatocellular carcinoma [9–11].

The impact of scan and reconstruction parameters on relevant imaging features is an important aspect of CRLM prognosis using CT imaging. For example, lowering the X-ray tube voltage may improve the contrast, better preserving texture details, but it also increases the image noise. On the reconstruction side, the kernel used to filter sensor data is a tunable factor that can reduce noise in the final image to varying degrees, but at the risk of over-smoothing details. Despite their importance, the scan and reconstruction parameters are heterogeneous across studies in the current literature. Both the standardization and optimization of imaging parameters for a given task and the robustification of crucial image features to varying conditions are necessary to draw consistent conclusions regarding different biomarkers and their relationship with patient outcomes [12–14].

Optimization of the imaging procedure to guide CRLM treatment requires extensive exploration of the parameter space. Furthermore, images are traditionally optimized for human observers, who may prefer noise and artifact suppression over sharp features. The rise of artificial intelligence (AI) in computer vision has accelerated a new paradigm of radiomics, where useful prognostic and diagnostic features, which are often inconspicuous to the human eye, are learned over a large dataset rather than pre-determined [15–20].

Filtered back projection (FBP) is the basis for the reconstruction used in all commercial CT scanners and is a fast, analytical conversion from the sensor domain to the image domain. However, information is partially lost during this transformation (e.g., reprojecting the reconstructed image into the sensor domain does not produce the original sinogram), increasing the risk of losing prognostic details. Meanwhile, extracting radiomic features directly from the raw data avoids this issue, but information on a local region of interest (ROI, e.g., a met) is distributed throughout the sinogram, yielding a high-dimensional, challenging problem [21]. As such, we propose using a high-frequency kernel for reconstruction, which results in images that appear noisier but are in closer agreement with the raw data.

**Fig. 1:**
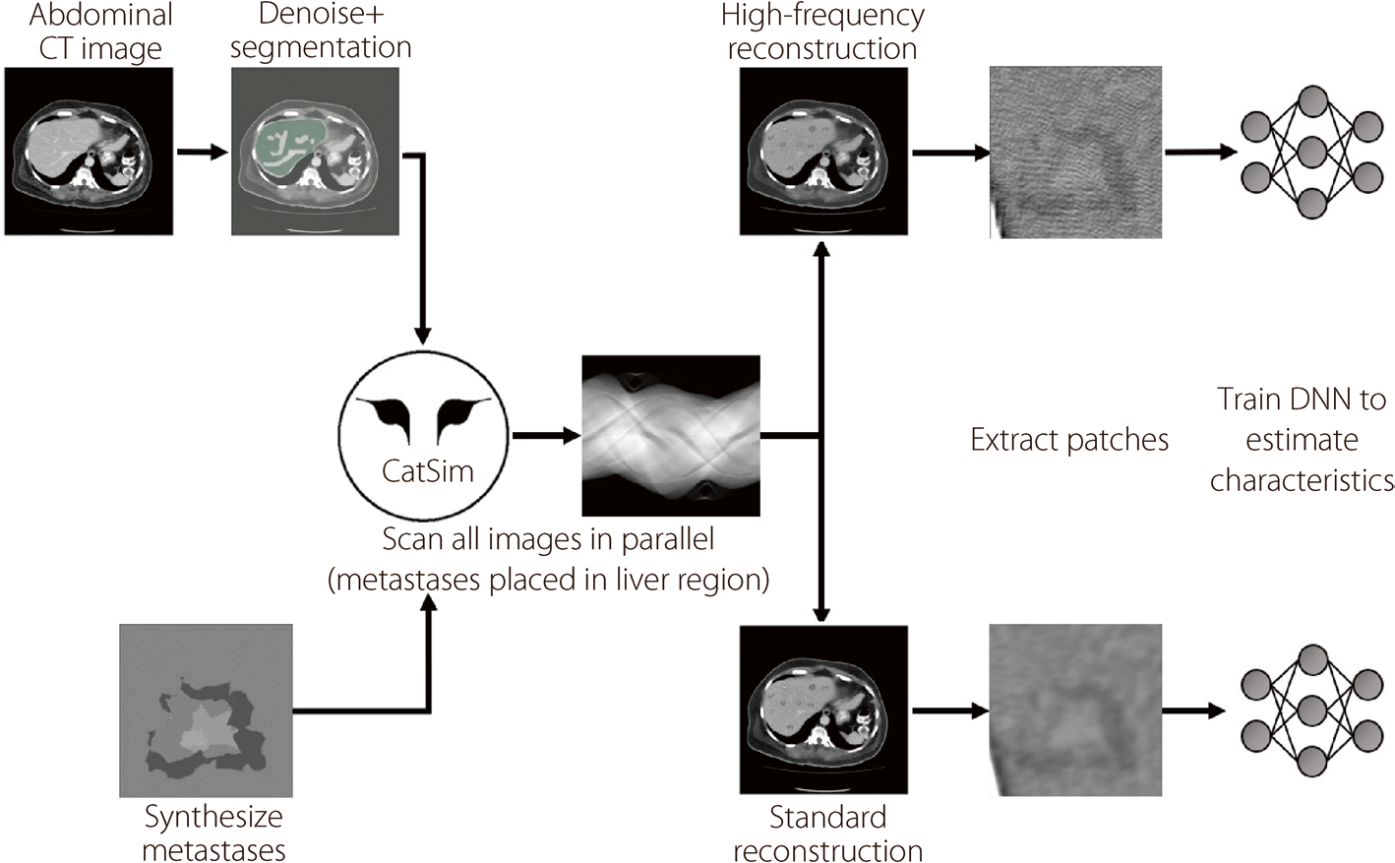
Flowchart of liver met simulation and radiomic analysis. One the left side, CT image backgrounds are denoised and segmented for liver regions, while met shapes are generated using a fractal method. The CT scans of backgrounds and mets are simulated in parallel using CatSim. The scan data are reconstructed using FBP with either a standard or high-frequency kernel; image patches of mets are extracted from the reconstructions and then used to train deep neural networks tasked with estimating the correct met characteristics

It is hypothesized that an AI observer can better recover met features from images reconstructed using a high-frequency kernel as opposed to the standard kernel. As it is impractical to initially evaluate this using a large cohort of patients, we propose a virtual imaging approach [22] to explore various imaging parameters in the context of CLRM. In this paper, we define a virtual imaging pipeline for simulating CT scans of liver mets with varying scan and reconstruction parameters and use this virtual imaging pipeline to compare radiomic performance using FBP reconstruction with either a standard or high-frequency reconstruction kernel. This approach is illustrated in Fig. 1.

Our met generation process employs a fractal generation method along with a post-smoothing operation that models a diverse distribution of realistic shapes with varying edge sharpness. Inside the mets, nested regions with varying grey scale are also generated to model the internal heterogeneity in density. As these mets are synthetic, they do not have specific associated prognoses; however, their exact characteristics (e.g., edge fractalness) are precisely known because these ground-truth images are used as input to the CT simulation. Consequently, they are useful for evaluating different scanning and reconstruction schemes because the ability of an imager to preserve these characteristics of virtual mets is likely associated with its ability to preserve clinically relevant features of real mets. In our experiments, we assessed the scheme’s ability to preserve characteristics using a deep neural network to recover these characteristics post-reconstruction.

## Methods

### Met synthesis

#### Random shape generation

Generating a diverse set of natural-appearing met shapes is a non-trivial task. Random initial met shapes are synthesized by generating vertices of a random fractal shape using an “infinite detail” method inspired by ref. [23] and then smoothing the shape with a moving average filter applied over the list of vertices. The vertex-defined shape is converted into a pixel map of values prior to simulation, providing a practical limit beyond which additional vertices between adjacent pixels are unimportant. Specifically, for fractal shape generation, six initial vertices defining a rough hexagon are initialized; the midpoints along each edge are then perturbed by random noise sampled from a uniform distribution that is scaled by the distance between the edge vertices for that mid-point and a roughness parameter, doubling the number of edges. The use of a distribution function and scaling argument provides a range of shapes, each with a different fractal dimension when measured. This process is repeated until the separation between vertices is less than the resulting inter-pixel spacing, creating more fractal detail with each iteration. To produce diversity in edge smoothness, the list of vertex coordinates is convolved with a moving average filter of random length up to half the number of vertices (a longer kernel produces a smoother shape). We also extend this technique to include an internal structure defining new shapes that are seeded with an early generation of surrounding shapes scaled to a smaller size that are allowed to evolve along a separate path but confined to reside within the surrounding shape. Table 1 summarizes and illustrates the different met parameters used for generation, as detailed in the following sections.

**Table 1:**
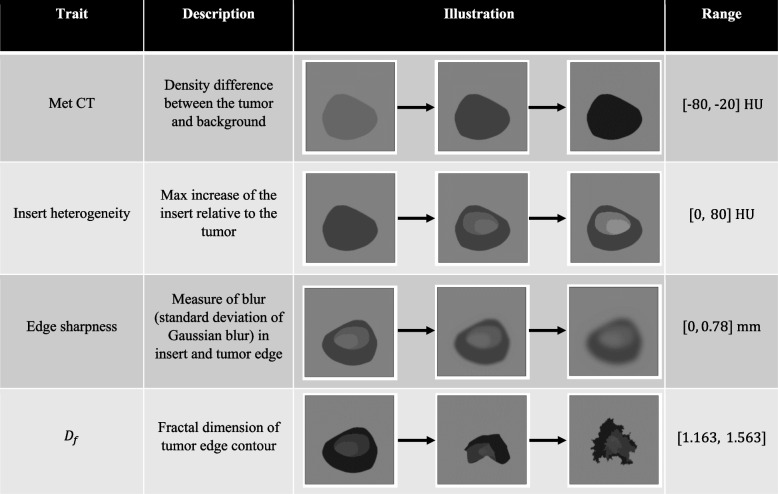
Description and illustration of met parameters and characteristics used in generation

#### Heterogeneity scaling and intensity scaling

**Fig. 2:**
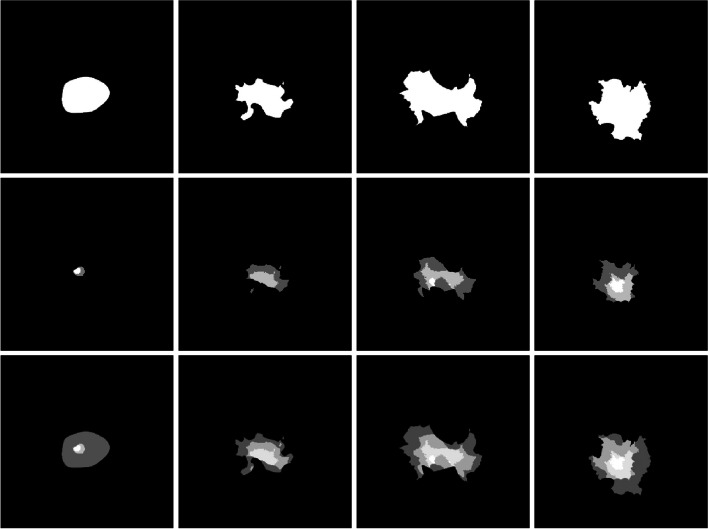
Samples of generated met shapes. Top row: met background shapes; Middle row: met insert shapes; Bottom row: overall met

The grayscale contrast (difference between met and background CT numbers) of each met is randomly sampled from a uniform distribution between -80 HU and -20 HU (Hounsfield Units). In addition to this homogenous base, a region of heterogeneity (referred to as the ‘insert’) is superimposed over each met. These inserts each consist of 2–3 sub-shapes, which are generated using the same fractal generation and smoothing method but fit within the met boundary. Examples of random met shapes and their insets are shown in Fig. 2. This insert is scaled such that the maximum difference between an insert point and the met background is uniformly sampled between 0 and 80 HU.

#### Fractal characterization

In addition to the generative parameters mentioned above, we characterize the jaggedness along the outer edge of each met with a fractal dimension. Met shapes are quantized as a $256\times 256$ bit map and then processed with an edge detector. The nuclear box-counting method is then used to calculate the fractal dimension of the edge images using box sizes of 1, 2, 4, 8, 16, and 32 pixels, with the fractal dimension being the average slope of the log–log plot of the box scale ${r}_{i}\in \{1, 2, 4, 8, 16, 32\}$, where $N(r)$ boxes are required to cover the contour [24]. In other words, fractal dimension ${D}_{f}$ is calculated by linear regression:

1 $$\begin{aligned}\left[\begin{array}{c}{D}_{f}\\ b\end{array}\right] &={(A}^{\top }{A)}^{-1}{A}^{\top }N \\ A&={\left[\begin{array}{ccc}-{\text{log}}{r}_{1}& \cdots & -{{\text{logr}}}_{6}\\ 1& \cdots & 1\end{array}\right]}^{\top },\\ N&={\left[\begin{array}{ccc}{\text{log}}N\left({r}_{1}\right)& \cdots & {\text{log}}N\left({r}_{6}\right)\end{array}\right]}^{\top }\end{aligned}$$

### Background preprocessing

**Fig. 3:**
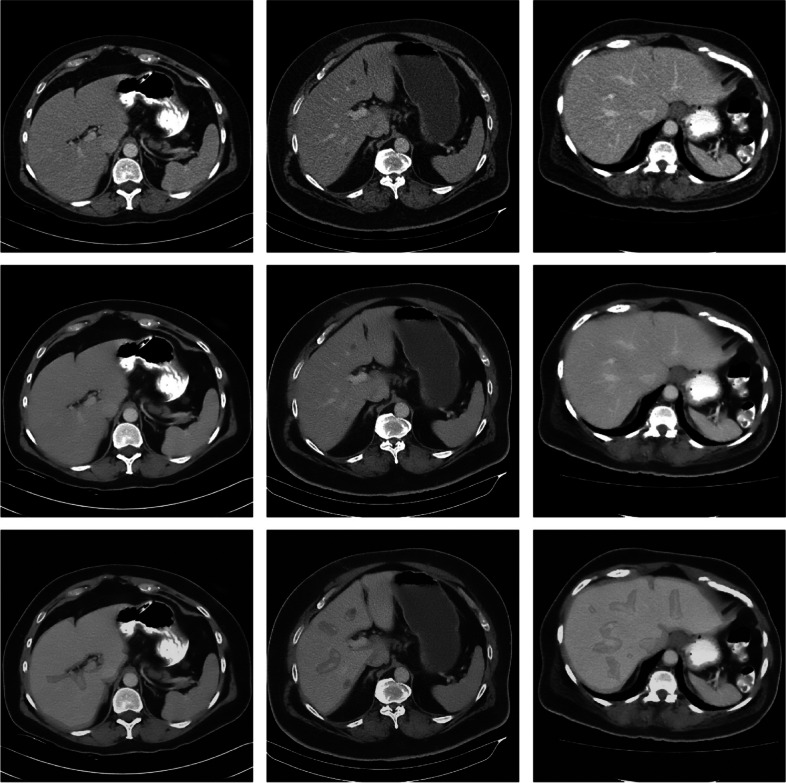
Sample images illustrating the clinical background image preprocessing. Top row: original clinical images; Middle row: denoised images; Bottom row: liver background segmentation

Contrast-enhanced CT scans of stage 1–3 colon cancer patients (no liver mets) were sourced from Kingston Health Sciences Centre (Queen’s University). Twenty $512\times 512$ slices with large liver regions were selected as image backgrounds. Because the pre-existing noise in these slices was compounded with noise added during the CT simulation, the images were pre-processed to reduce noise in the input images. The “Reduce Noise” filter from Adobe Photoshop Elements 11 was used to denoise the clinical background images. Subsequently, the liver region of each slice was manually segmented, excluding blood vessels, cysts, and other confounding structures (Fig. 3), to identify regions suitable for synthetic met insertion (mets should be positioned inside the liver and not coincide with other structures).

### Simulation

CatSim was used to simulate 2D scans of image backgrounds with superimposed synthetic mets [25, 26]. The image backgrounds were converted into water density maps based on their CT number. Synthetic mets were randomly positioned within the liver segmentation map in $25\ \mathrm {mm}\times 25\ {\text{mm}}$ non-overlapping patches. Rather than superimposing the mets and clinical backgrounds in the image domain, which would limit the met detail to the resolution used for the clinical background images, the images were reprojected separately and superimposed in the sinogram domain. As such, the mets were simulated at a much higher resolution (voxel size 0.156 mm) than the clinical backgrounds (voxel size 0.68 mm to 0.82 mm). Ten to twelve mets were superimposed on each background. Although the same backgrounds were used in multiple scans, the met placement varied between scans, resulting in diverse image patches. The scanning parameters were set to mimic those of the Lightspeed VCT scanner (GE HealthCare): 1.0239 mm detector cell pitch, 888 detector cells, $984$ views, $140 {{\text{kV}}}_{{\text{p}}}$ X-ray tube voltage. The source-to-isocenter distance and source-to-detector distance were 538.52 mm and 946.75 mm, respectively.

### Reconstruction

**Fig. 4:**
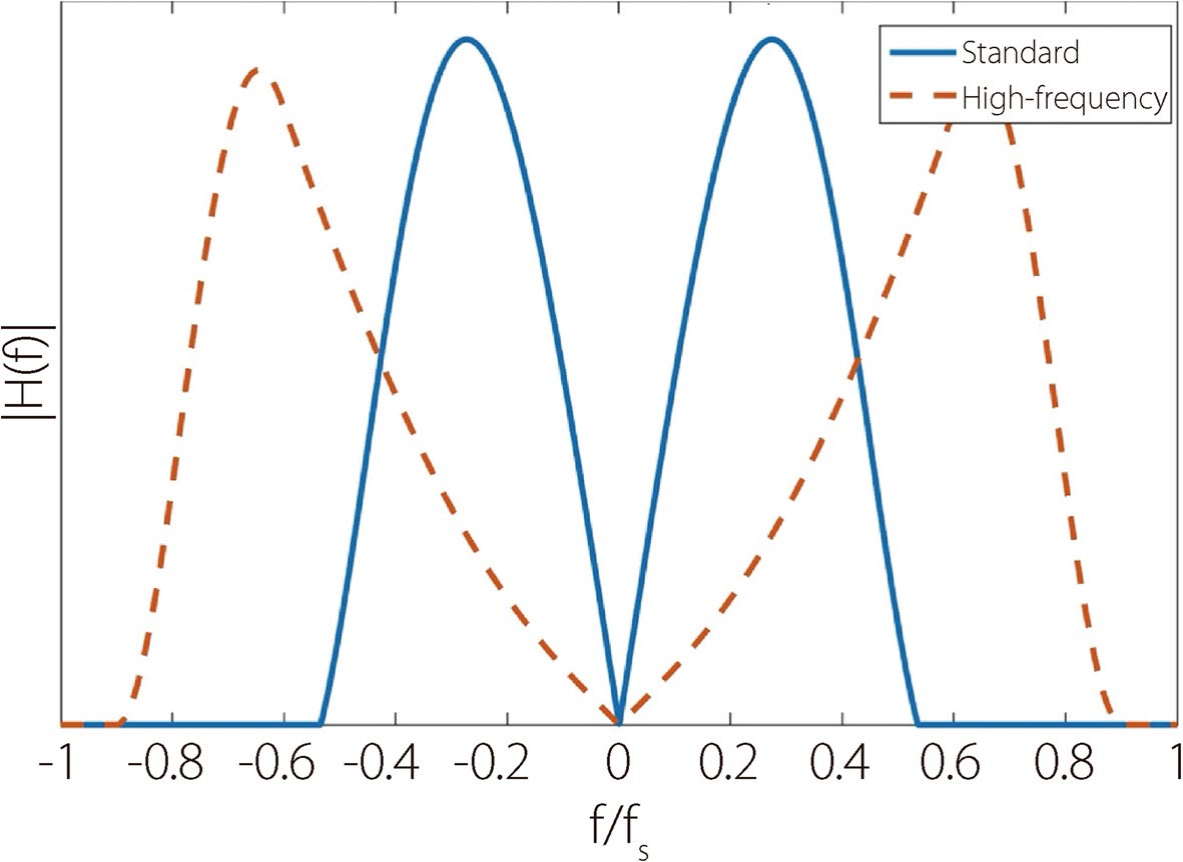
Comparison of the absolute frequency responses $\left|H\left(f\right)\right|$ between the standard and high-frequency reconstruction kernels. ${f}_{s}$ is the sampling frequency

Each scanned slice was reconstructed twice using FBP with either the ‘Standard’ or ‘Edge’ (high-frequency) GE HealthCare product kernels. Figure 4 illustrates the absolute frequency responses of the two kernels for comparison. All images were reconstructed with a 40 cm field-of-view and 0.2 mm voxel size.

### Characterization studies

The goal of the virtual imaging trial was to evaluate how accurately heterogeneity, edge sharpness, and edge fractal dimension can be characterized, as defined in Table 1. We found that the edge sharpness and fractal dimension could not be easily evaluated within the same dataset, as blurring the edge of a met would destroy the original fractal dimension of the shape. In addition, we observed that the preservation of these features was highly sensitive to noise level during the simulation. Consequently, the following four studies were conducted separately to broadly capture feature preservation effects:

1. Noiseless/no-blur: Evaluated for insert heterogeneity and fractal dimension.
2. Noiseless/blurred: Evaluated for edge sharpness.
3. Noisy/no-blur: Evaluated for insert heterogeneity and fractal dimension.
4. Noisy/blurred: Evaluated for edge sharpness.

No Gaussian blurring was applied to the met shapes in the no-blur studies. Poisson noise was simulated with a tube current of 600 mA and a rotation time of 1 s for the noise studies. The signal-to-noise ratio of these scans was calculated to be $48.4143\pm 0.8139$ dB. No noise was inserted in the noiseless experiments. Ten thousand mets were generated and used in each simulation study. Total simulation time was approximately 36 h on a Linux system with a 12@Intel(R) Core™ i7-5930 K processor and 100 GB DDR4 RAM (although shared with other jobs running on the same server).

### Network architecture and training

**Fig. 5:**
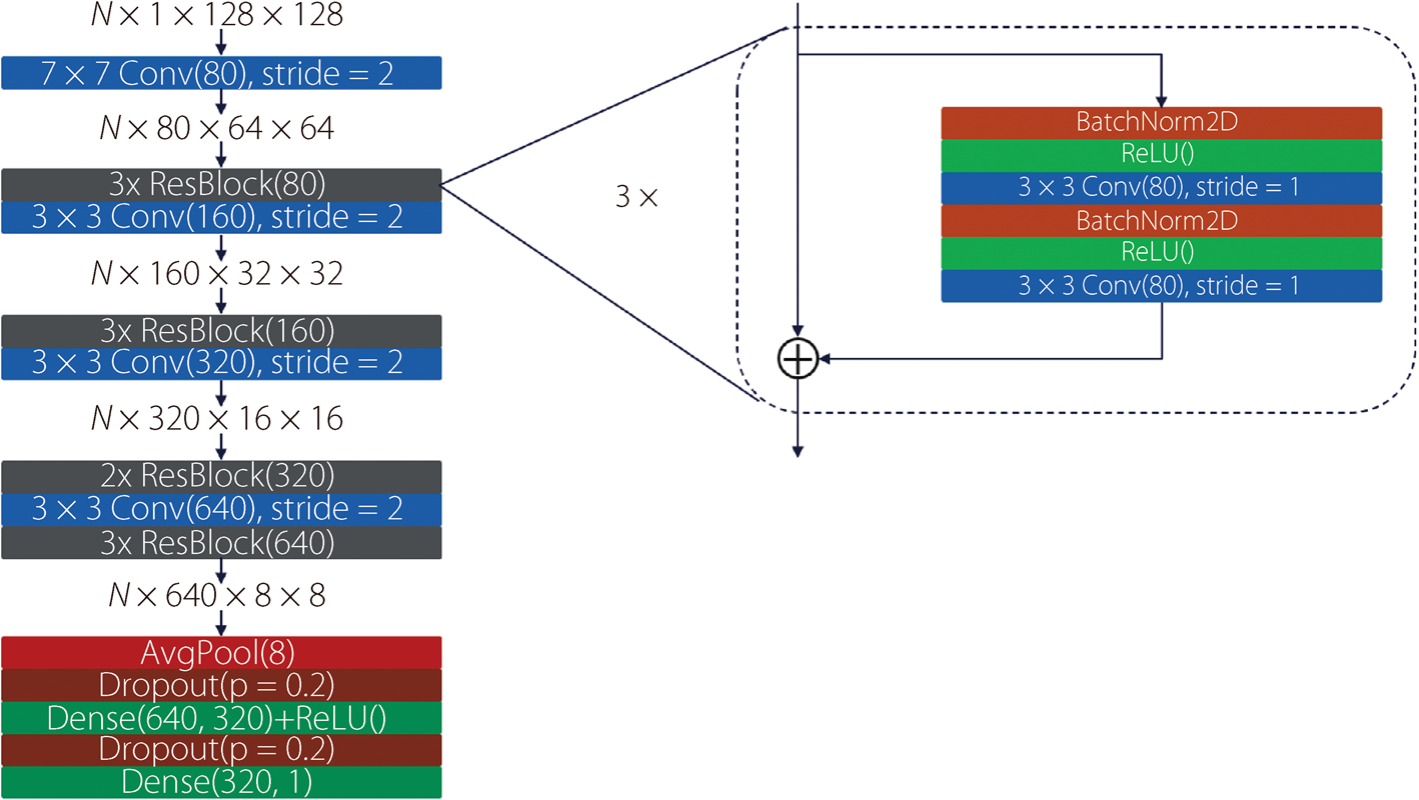
Diagram of the deep neural network architecture used for all characterization tasks. Conv(n) signifies a convolutional layer with *n* output filters

The goal of the characterization study was to use a deep neural network to estimate the true met parameters from reconstructed images. Reconstructions were cropped to create $128\times 128$ image patches centered on each met. PyTorch version 1.110 was used with an NVIDIA Titan RTX and CUDA 10.2 for deep learning training and testing. The network architecture was roughly based on ResNet V2 [27, 28]. Optuna was used to search the hyperparameter space [29]. The final architecture is shown in Fig. 5.

Different models were trained and evaluated for each characteristic. Edge fractalness and insert heterogeneity were evaluated using the datasets without blur, while edge sharpness was evaluated on the dataset with blur. Each evaluation used a 90/10 training/validation split. Random horizontal and vertical reflections, along with random image rotations, were used for eight-fold training data augmentation. The data and labels were normalized. The Adam optimizer was used with a learning rate of $4\times {10}^{-5}$ and mean-squared error loss function.

Each network was trained using a batch size of 40 samples over 120 epochs. At the end of each epoch, a “bias adjustment” was performed, where the parameters of the final dense layer were adjusted using a globally computed linear regression (across the entire training dataset). This adjustment corrects any small bias that could result from sampling only a small batch, thereby helping the models to converge. After training, we calculated the errors in each validation sample as well as the concordance correlation coefficient (CCC) between the validation predictions and labels.

## Results

### Reconstructions

**Fig. 6:**
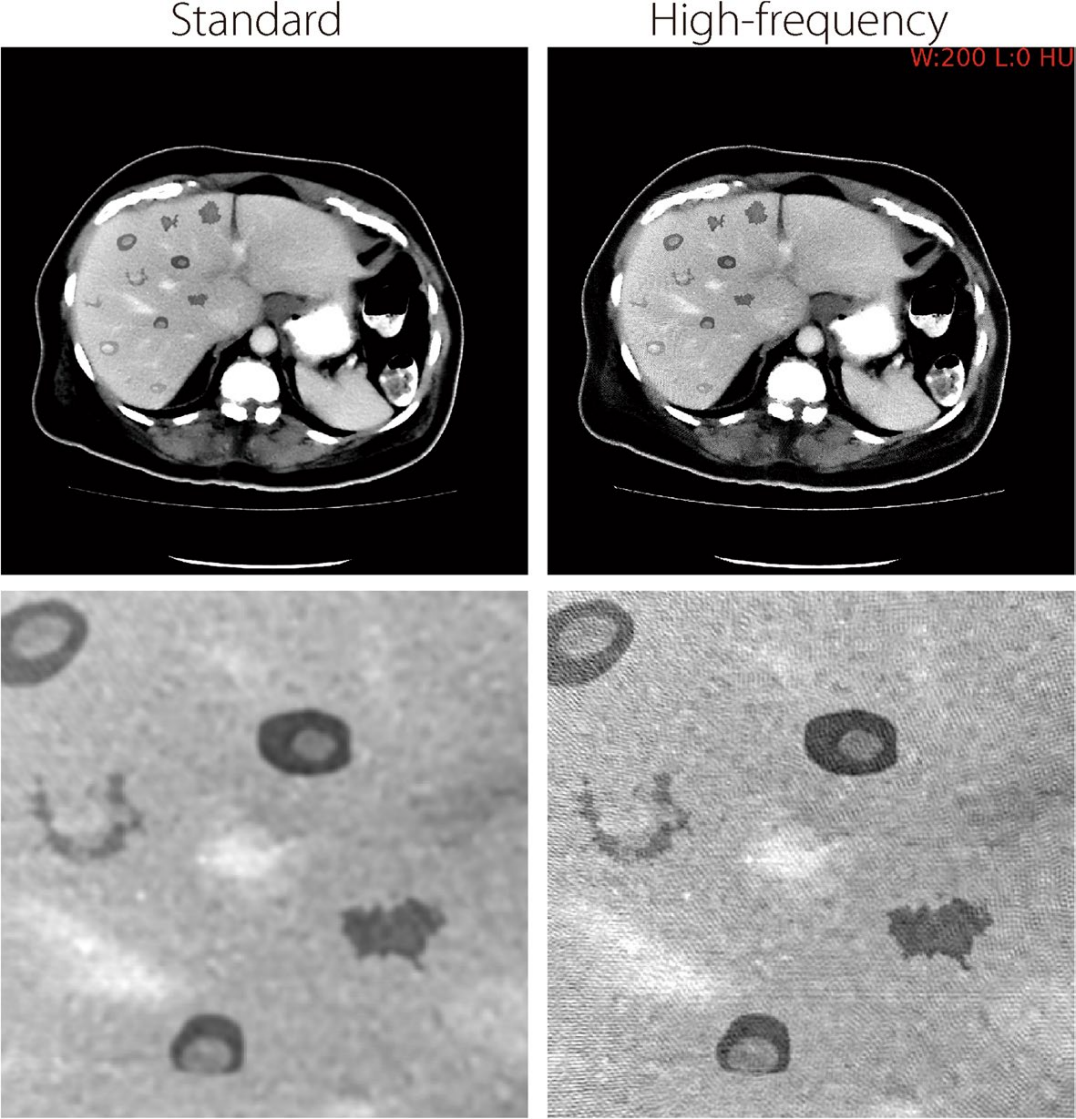
Sample abdominal reconstruction from the noiseless/no-blur study using standard (left) and high-frequency (right) filtering

Figure 6 illustrates full field-of-view reconstructions of a liver cross-section using both standard and high-frequency filtering, along with a magnified window of a met region. The mets were randomly placed across the liver region while avoiding overlap between mets other structures, such as blood vessels. In general, high-frequency images yield sharper edges but at the expense of increased aliasing artifacts.

**Fig. 7:**
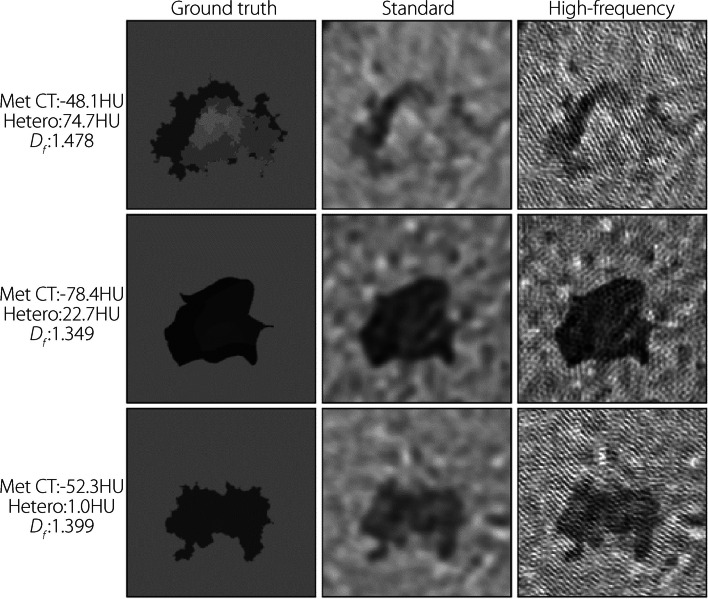
Samples of met patches pre-simulation (left) and after reconstruction using standard (middle) and high-frequency (right) kernels for the noiseless/no-blur study. Characteristics are on the left. Window and level are 200 and 0 HU, respectively, for all images

Figure 7 shows several example mets without noise or blur, with varying CT number, heterogeneity, and edge fractalness. The fractal edges of the shapes are noticeably better defined in the high-frequency images, while many edge details from the original met are absent in the standard reconstruction. Internal heterogeneity is generally detectable, although the background texture can obfuscate this trait.

**Fig. 8:**
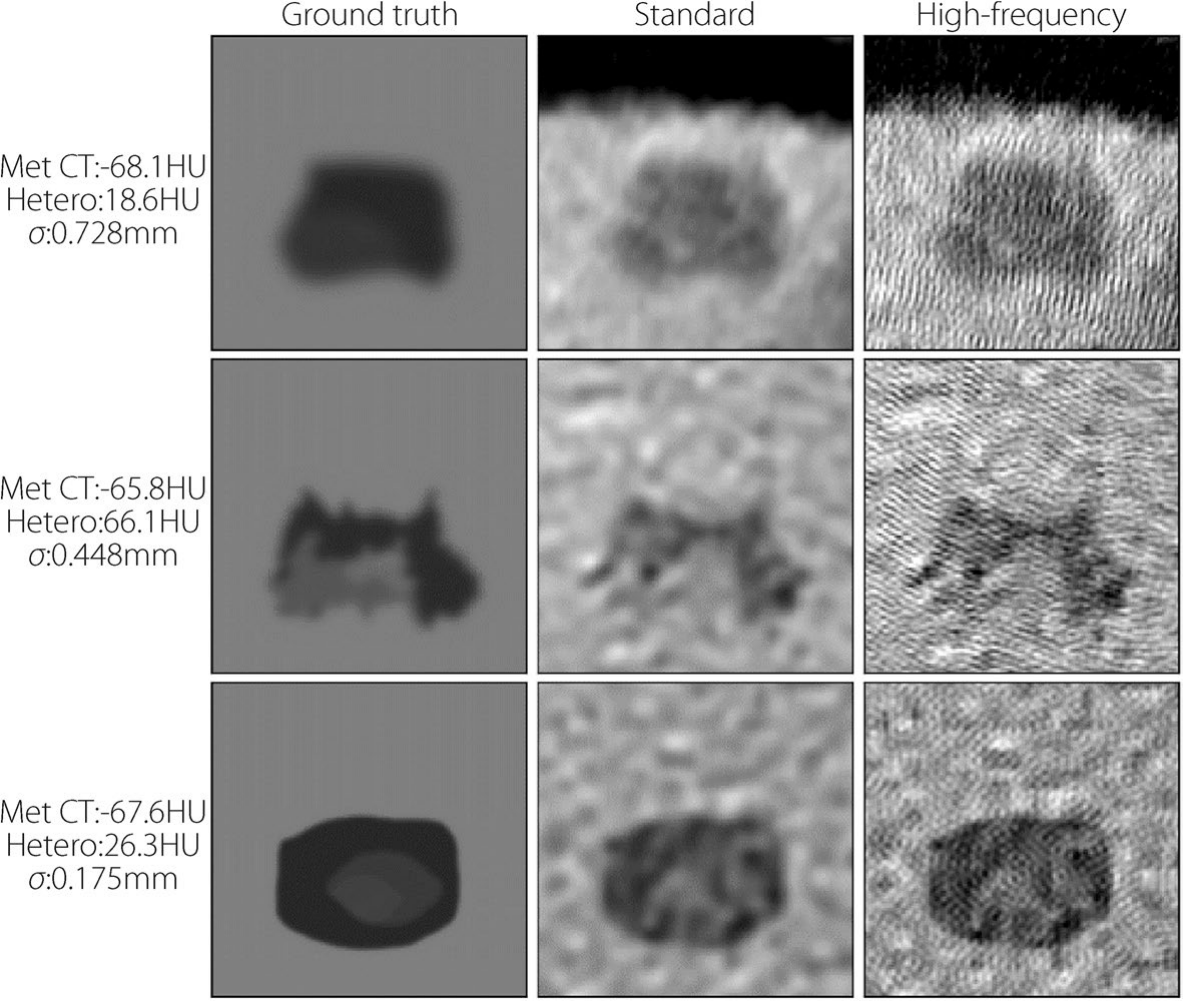
Samples of met patches pre-simulation (left) and after reconstruction using standard (middle) and high-frequency (right) kernels from noiseless/blurred study. Characteristics are on the left. Window and level are 200 and 0 HU, respectively, for all images

Similarly, Fig. 8 illustrates several examples of mets without noise and with varying amounts of blur (or edge sharpness). The degree of edge sharpness (blur) appears easier to distinguish in the high-frequency reconstructions, especially in instances of minimal blur, because the standard reconstruction adds its own blur to the image.

**Fig. 9:**
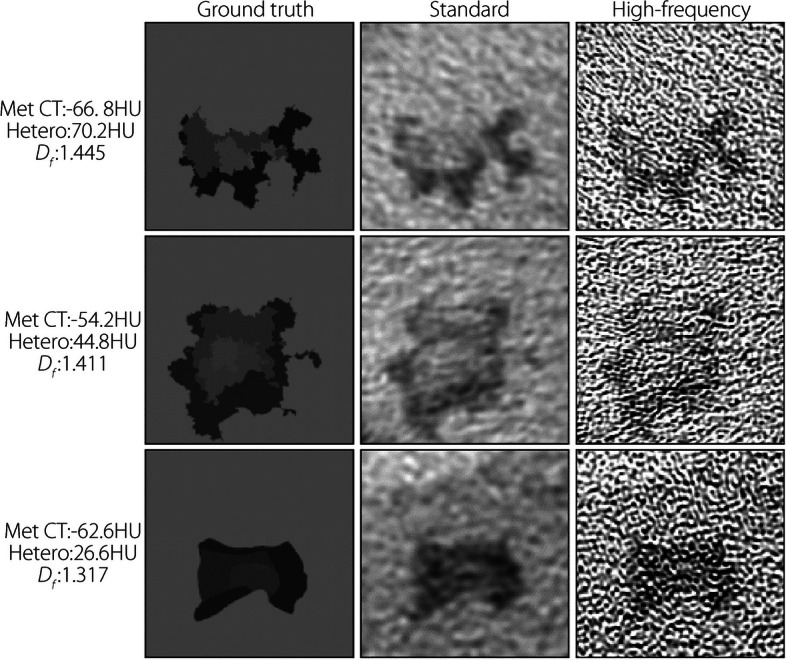
Samples of met patches pre-simulation (left) and after reconstruction using standard (middle) and high-frequency (right) kernels from the noisy/no-blur study. Characteristics are on the left. Window and level are 200 and 0 HU, respectively, for all images

**Fig. 10:**
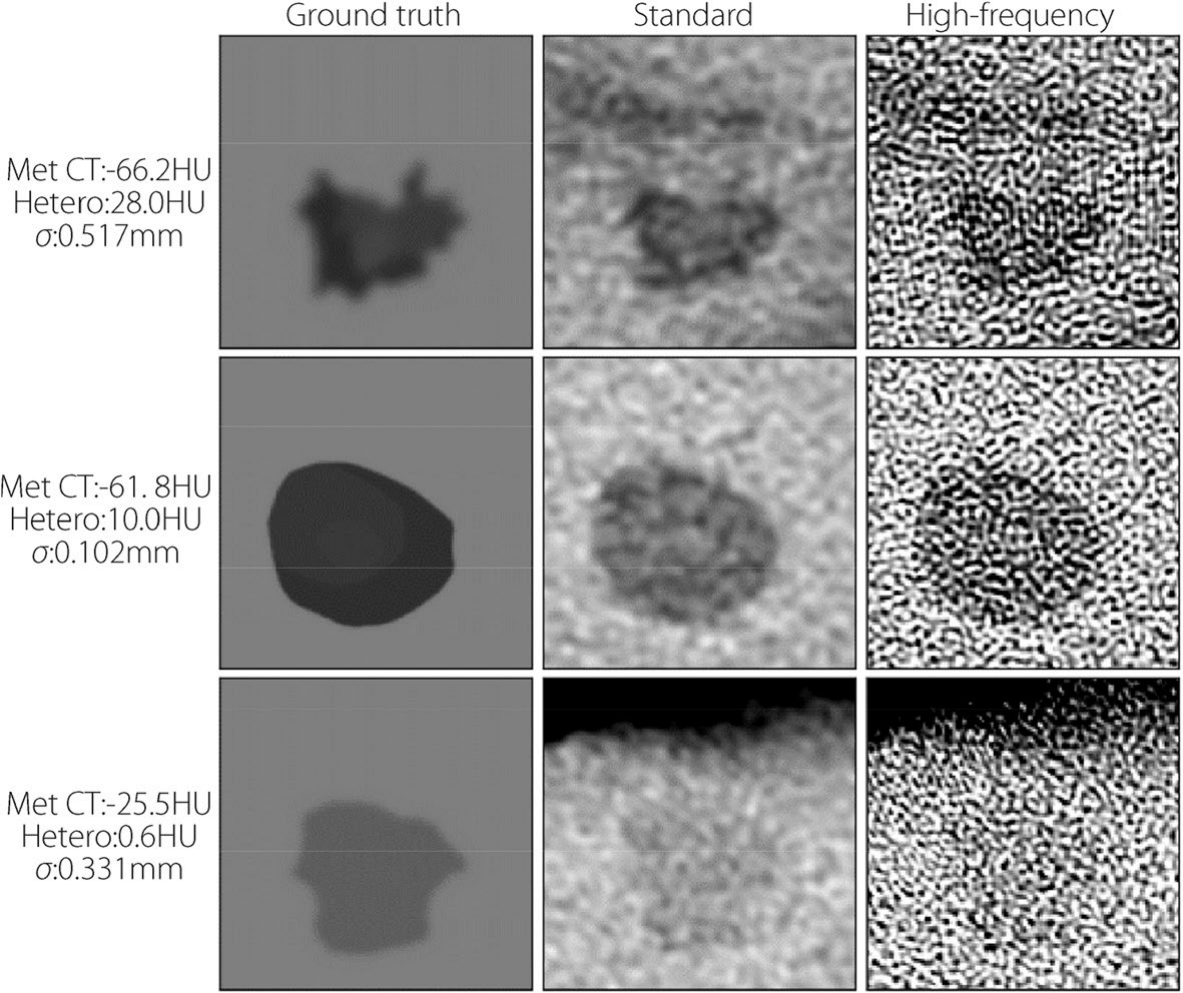
Samples of met patches pre-simulation (left) and after reconstruction using standard (middle) and high-frequency (right) kernels from the noisy/blurred study. Window and level are 200 and 0 HU, respectively, for all images

Figures 9 and 10 show the corresponding sample patches for noisy no-blur and blurred images. Edge sharpness and details are difficult to detect in both reconstructions, and the added noise particularly affects the appearance of the high-frequency images, with edge details being heavily degraded. In these noisy instances, mets with lower density and heterogeneity are easier to detect because they are more distinct from their backgrounds.

### Deep characterization performance

**Fig. 11:**
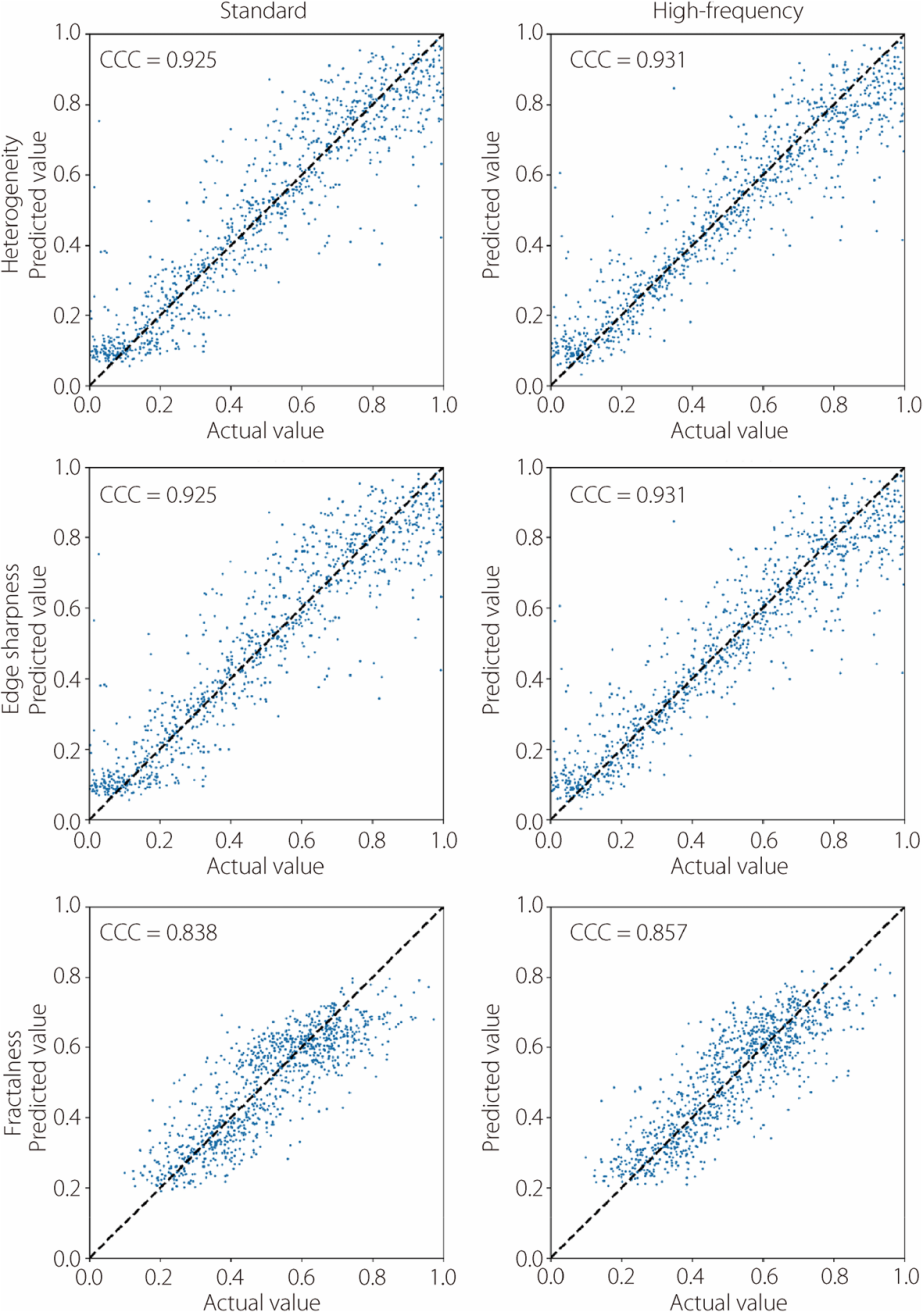
Actual *vs* predicted regression plots on validation dataset in the noiseless studies for heterogeneity (top), edge sharpness (middle), and fractal dimension (bottom). All label ranges are normalized to [0, 1]

**Fig. 12:**
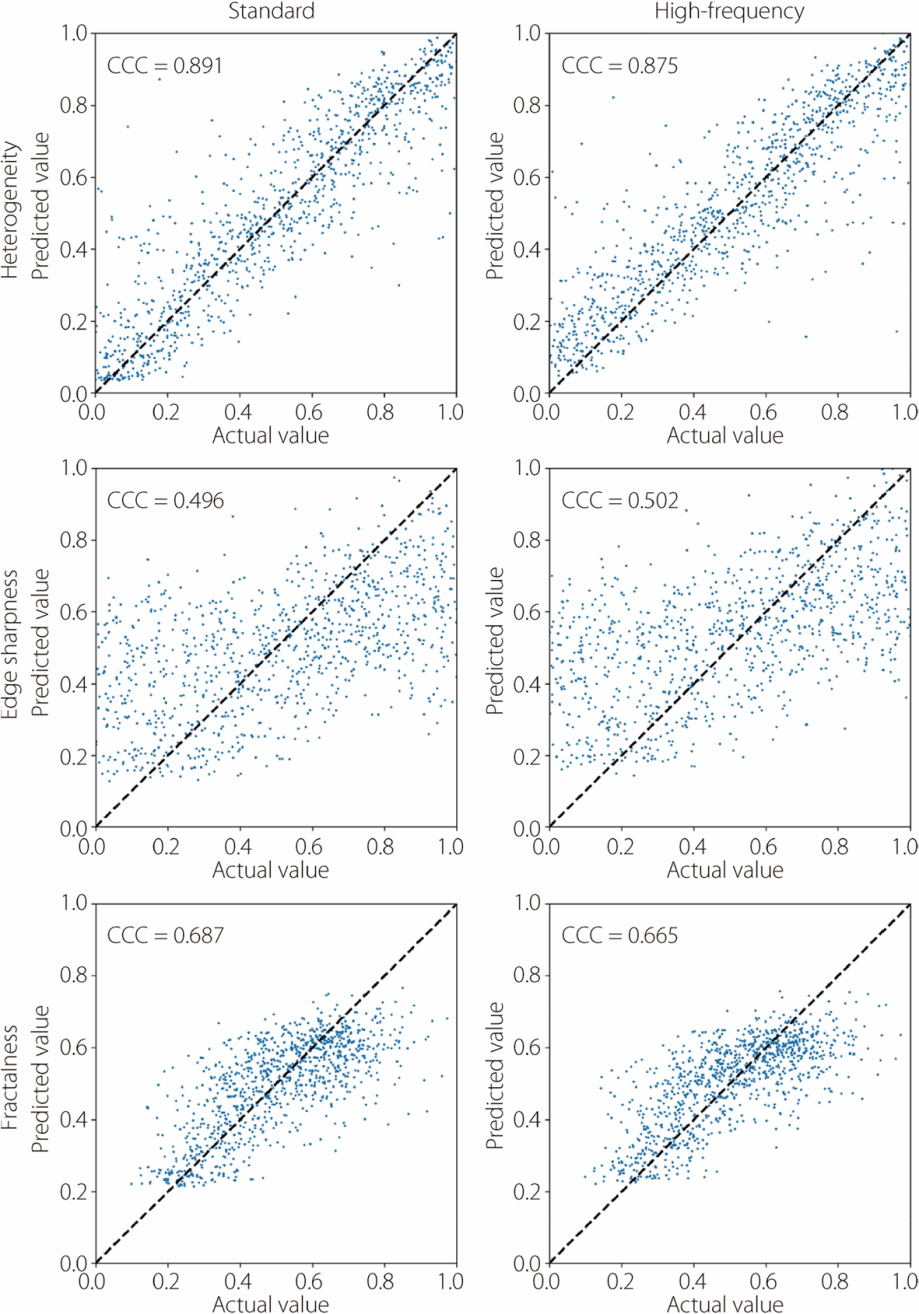
Actual *vs* predicted regression plots on validation dataset in the noisy studies for heterogeneity (top), edge sharpness (middle), and fractal dimension (bottom). All label ranges are normalized to [0, 1]

**Fig. 13:**
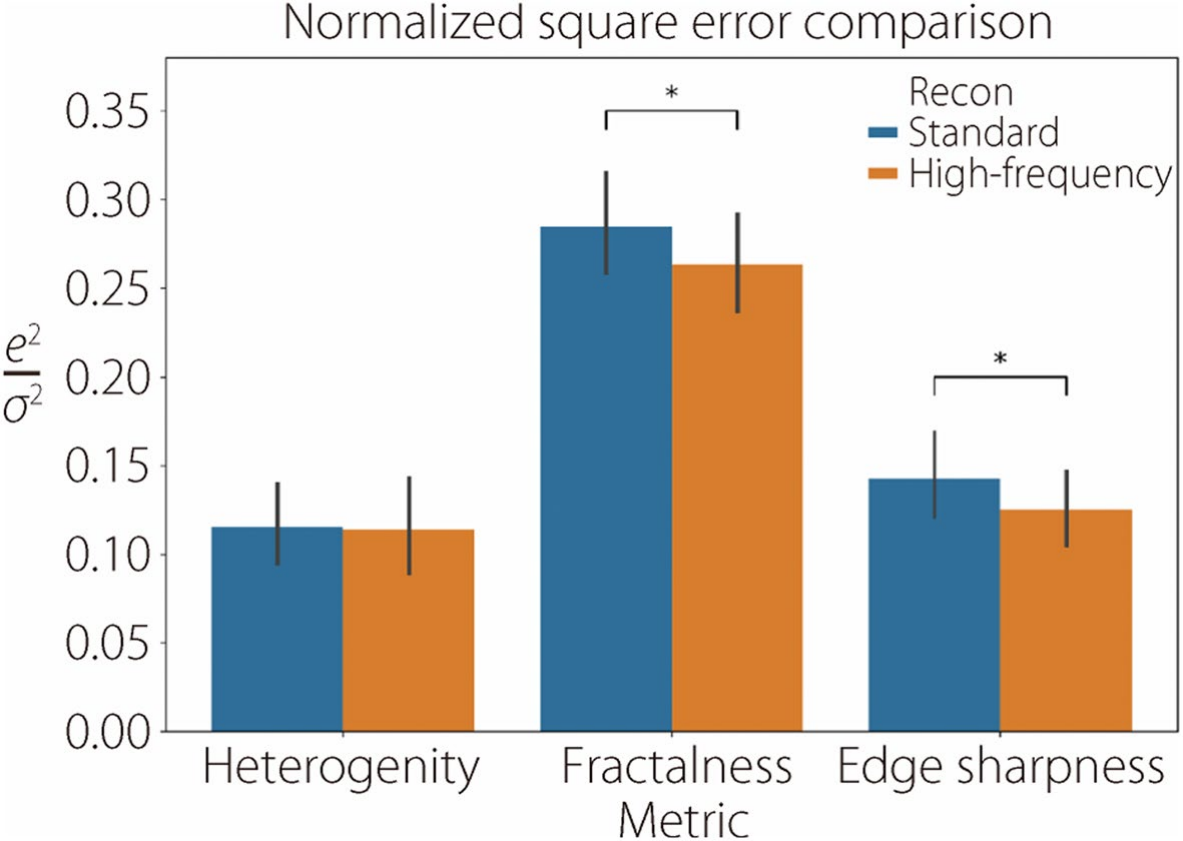
Average squared error (normalized by label variance) of deep characterization on validation data in the noiseless studies. Error bars represent 95%CI. * denotes a statistically significant difference (*p* < 0.05)

**Fig. 14:**
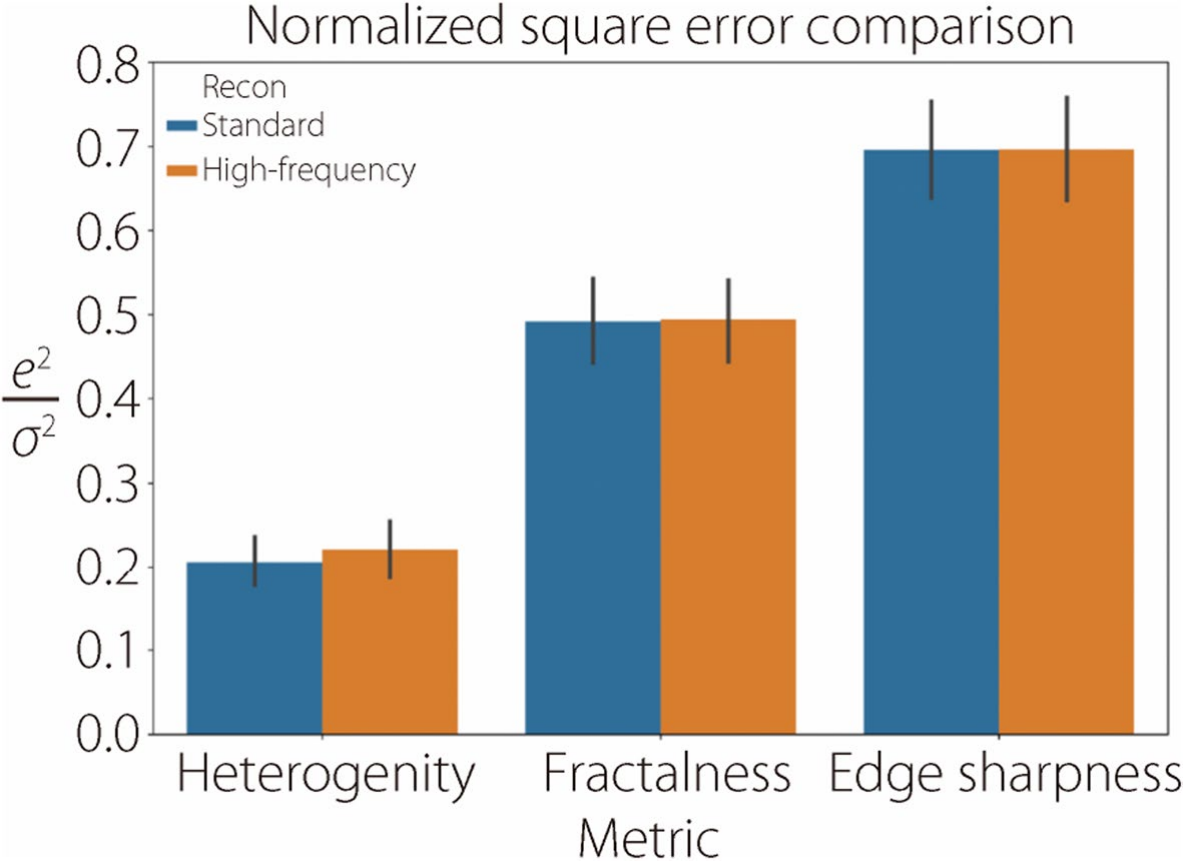
Average squared error (normalized by label variance) of deep characterization on validation data in the noisy studies. There are no significant differences between standard and high-frequency reconstructions

The performance of deep characterization using standard and high-frequency reconstructions is reported across all studies by visualizing scatter plots of actual *vs* predicted labels on the validation datasets. Figure 11 shows these plots for insert heterogeneity, edge sharpness, and fractal dimension in the noiseless studies, while Fig. 12 shows the same results for the noisy studies. CCC are also included in the evaluation. Heterogeneity is characterized comparably between the standard and high-frequency methods, but fractalness and edge sharpness show superior fits with the high-frequency reconstruction (Fig. 11). This superior fit is largely due to samples with high fractalness, where the standard method tends to underestimate this trait, and samples with sharp edges (low blur), where the standard method overestimates the blur. This is likely due to the standard kernel’s degradation of high-frequency details, which is critical for proper characterization in these instances. However, these regressions are much worse when noise is introduced (Fig. 12). Figure 13 summarizes the average squared error of the validation label prediction for each instance. Two-tailed paired *t*-tests were performed, showing a significant difference in squared error when using standard *vs* high-frequency reconstruction for predicting edge sharpness ($\alpha =0.012)$ and fractal dimension ($\alpha =0.049)$. No significant differences were observed in any of the characteristics in the noisy studies (Fig. 14). A comparison of noiseless and noisy performance yielded significant differences for all three metrics with either reconstruction method.

## Discussion

Generating a realistic but diverse population of mets for image simulation is an outstanding problem, and the increasing complexity and variety of imaging systems have increased the demand for virtual clinical trials [22]. Previous studies explored complex solutions to this topic by generating adequate data for AI image analysis [30, 31]. Although our approach does not perfectly model real liver metastases, it can efficiently produce a diverse set of varying features that is sufficiently large and representative to explore the original hypothesis. Furthermore, unlike methods that superimpose shapes over reconstructed images, our approach generates shapes at a high resolution *prior* to scanning and reconstruction. As such, the pre-scan shape information is known precisely, allowing the evaluation of how different imaging and reconstruction settings preserve or degrade these features.

Placing as many mets within a background as possible without overlapping (Fig. 6) reduces the number of scans required to generate sufficiently large datasets. The overall size of each met was restricted due to this consideration. This highlights a tradeoff between simulation speed and magnitude/diversity in met size.

Although this simulation approach is advantageous for investigating the impact of higher-resolution imaging methods for more accurate characterization of representative but hypothetical radiomics features, this study did not aim to train or evaluate a radiomics approach for predicting clinical outcomes from real patient images. By nature, the generated mets have well-defined anatomical characteristics, but clinical labels differentiating met behavior and malignancy inherently cannot be generated in this manner. However, as previously discussed, many anatomical features of mets, such as fractalness and texture, are related to their clinical classification. Consequently, imaging methods that better preserve these features are assumed to contain more clinical information; however, a direct evaluation on a real, clinically labelled dataset is required to validate this. As such, given its efficiency, the proposed pipeline is best suited for representative but hypothetical experiments (e.g., optimizing parameters that extract targeted features, such as fractalness and texture), rather than clinical experiments, which require real scans specific to a medical task. Hence, future studies should test whether high-frequency kernels can produce more accurate prognostic labels for liver mets in low-noise situations.

Compared with the standard kernel, high-frequency kernel reconstructions feature aliasing-like noise patterns (Figs. 7 and 8). This is expected because the high-frequency kernel preserves frequencies higher than those classically permitted by the Nyquist theorem. Standard filtering removes this high-frequency noise but inevitably destroys the high-frequency features of the underlying signal in this process.

The results shown in Figs. 11 and 13 indicate that deep learning methods can more accurately recover high-frequency characteristics, such as edge sharpness and fractal dimension, compared to standard kernel reconstructions. Although the noise patterns from high-frequency filtering are less appealing to human observers, sufficiently trained deep learning models can leverage the underlying high-frequency signal that is preserved in high-frequency filtering. This advantage is apparent from the noiseless studies, but prediction of high-frequency features is considerably degraded when Poisson noise is introduced (Fig. 12). Unlike the aforementioned aliasing noise, which is an artifact of sampling and reconstruction, Poisson noise is innate to raw data and cannot easily be circumvented. As such, neither reconstruction method shows a distinct advantage, as the raw data itself limits the recoverability of high-frequency features, as opposed to the reconstruction method (Fig. 13). The significant difference in performance between noiseless and noisy data, which is observed in all metrics with either reconstruction, reinforces the importance of noise level in characterizing these features. Future research should investigate the use of high-frequency reconstruction in low-noise tasks, such as imaging over a small ROI where high resolution is required. High-frequency reconstruction can also be used in conjunction with downstream data-driven processing, such as other analysis tasks or deep image denoising [32–34].

In future work, FBP with alternative kernels should be compared to iterative reconstruction within our framework. Iterative reconstruction algorithms are generally more robust against common CT image artefacts and quantum noise [35]. Therefore, iterative reconstruction could potentially better preserve the met features assessed in these experiments. However, one associated pitfall is the extended time requirement for iterative methods; testing iterative reconstruction over many scans with our pipeline would likely take much more time. An ideal target from such experiments could be an optimized filter kernel that produces results close to those of iterative reconstruction while maintaining the efficiency of FBP.

## Conclusions

In this study, we investigated FBP with a high-frequency kernel, which better preserves scan data, and compared it with a standard filter kernel for feature preservation of virtual CT CRLM. The virtual imaging framework used to explore this quickly generated a diverse population of met shapes via a fractal-based method, which are superimposed on real clinical backgrounds. This method was able to simulate 10,000 CT met images for both high-frequency and standard kernel reconstructions in approximately 36 h with a 12@Intel(R) Core™ i7-5930 K processor. Our deep radiomics analysis suggests that when image noise is sufficiently low, high-frequency filter reconstruction is superior for preserving high-frequency features, such as edge fractalness and sharpness, and might reasonably be expected to better discriminate alternative image-based metrics considered for diagnostic purposes. Future studies should expand these simulation methods to improve clinical translation and add more features, such as complex texture variations. Additionally, future work should investigate high-frequency reconstruction in low-noise, high-resolution imaging applications and data-driven image tasks, such as deep denoising and further analysis.

## Data Availability

The datasets used and generated in this study are not publicly available at this time as they contain sensitive information in the form of real CT images from patients at Kingston Health Sciences Centre (Queen’s University).

## Abbreviations

CT: Computed tomography
CRLM: Colorectal liver metastases
FBP: Filtered back projection
CCC: Concordance correlation coefficient
AI: Artificial intelligence
met: Metastasis
ROI: Region of interest
